# Carnosol Maintains Intestinal Barrier Function and Mucosal Immune Homeostasis in DSS-Induced Colitis

**DOI:** 10.3389/fnut.2022.894307

**Published:** 2022-05-24

**Authors:** Xiang Xu, Gao Zhang, Kun Peng, Yanping Gao, Jinxia Wang, Caiping Gao, Chong He, Fang Lu

**Affiliations:** ^1^Clinical Immunology Translational Medicine Key Laboratory of Sichuan Province, Sichuan Provincial People's Hospital, University of Electronic Science and Technology of China, Chengdu, China; ^2^Department of Gastroenterology, Sichuan Provincial People's Hospital, University of Electronic Science and Technology of China, Chengdu, China

**Keywords:** ulcerative colitis, experimental colitis, carnosol, intestinal epithelial cells, endoplasmic reticulum stress

## Abstract

Ulcerative colitis (UC) is a chronic inflammatory disease, characterized by recurrent flares of mucosal inflammation, which is limited in the colon and rectum. Compromised epithelial barrier functions have been indicated in the initiation of UC. Carnosol (CA), a natural active ortho-diphenol diterpene compound, is one of the active ingredients in plants such as rosemary and sage. The anti-inflammatory and anti-oxidative effects of CA have been reported in several animal models, but its effect on mucosal inflammation remains elusive. We established a mouse experimental colitis model characterized by epithelial barrier destruction using dextran sulfate sodium (DSS). CA was intraperitoneally administrated. Flow cytometry was performed to determine phenotypes of intraepithelial lymphocytes and lamina propria cells. qRT-PCR was used for gene expression. ER stress in the colon was determined by immunofluorescence staining and qRT-PCR. Thapsigargin was used to induce ER stress in HCT-116 cells *in vitro*. We found CA significantly alleviated DSS-induced colitis in mice marked by relieved clinical symptoms and colonic pathological damage. Inflammatory cell infiltration and cytokine expression in the colon were suppressed by CA during colitis. Furthermore, CA restored epithelial barrier functions and intestinal intraepithelial lymphocyte (IEL) homeostasis in mice with DSS insults. Mechanistically, we induced endoplasmic reticulum (ER) stress in HCT-116 cells (an intestinal epithelial cell line) with thapsigargin, and CA reversed this effect. In addition, we collected inflamed mucosal biopsies from 23 patients with UC, and cultured overnight with or without CA, showing CA significantly reduced expression of ER stress signaling molecule and pro-inflammatory agents. Our data demonstrate that CA acts as an effective drug for experimental colitis and maintains proper epithelial barrier functions via suppressing epithelial ER stress, providing new evidence that CA might be a promising therapeutic candidate for UC.

## Introduction

Inflammatory bowel disease (IBD) has been recognized as a chronic inflammatory disease of the digestive tract. Over the past decades, studies show that the incidence and prevalence of UC have increased worldwide, leading to a substantial healthcare burden (1, 2). Ulcerative colitis (UC) is one of two clinical types of IBD and characterized by a typical natural course with recurrent flares of mucosal inflammation, which is limited in the colon and rectum. Although multiple factors have been implicated in the pathogenesis of the disease, such as immune responses, mucosal barrier, antimicrobial defense, and gut microbiota (3), the exact molecular mechanisms underlying the induction and development of UC remain largely unknown.

Carnosol (CA), a natural active ortho-diphenol diterpene compound, is one of the active ingredients in plants such as rosemary and sage (4). It is reported that CA has many biological activities such as anti-tumor, anti-inflammation and anti-oxidation (4, 5). For example, CA can inhibit tumor cell proliferation in diseases such as breast cancer (6), colon cancer (7), and prostate cancer (8). In addition, CA has been identified as a potent antioxidant and implicated in the treatment of various disease. For instance, CA inhibits AKT/mTOR, MAPK, NF-κB and other signaling pathways, and significantly down-regulated the expression of oxidative stress effector molecules such as COX-1/2, NO and iNOS (5). In diabetic microangiopathy, CA alleviates vascular endothelial cell damage by reducing the production of reactive oxygen species and maintaining tight junctions between endothelial cells (9). Lee et al. reported that CA effectively inhibited endotoxin-induced nitric oxide production and down-regulated the expression levels of pro-inflammatory cytokines in the peripheral blood of mice with odorous dermatitis, including TNF-α and IL-1β (10) As for their effect on regulating autoimmune disease, a recent study on experimental autoimmune encephalomyelitis (EAE), a mouse model of autoimmune disease of the central nervous system, demonstrated the anti-inflammatory effect of CA. Mice treated with CA were less susceptible to EAE induction, characterized by significantly lower inflammatory lesions and decreased inflammatory cell infiltration (11). However, how CA regulates intestinal inflammation and whether it plays a role in maintaining epithelial barrier functions remain unclear.

In the current study, we found that CA exerted a potent anti-inflammatory function in dextran sulfate sodium (DSS)-induced mouse colitis. Mice administrated with CA showed remarkably alleviated colonic inflammation after DSS insults, marked with less disrupted IEC barrier function and reduced inflammatory cell infiltration. Additionally, we found that CA down-regulated epithelial endoplasmic reticulum (ER) stress in colitis. Our data thereby demonstrate a potential role of CA in the treatment of IBD and the potential mechanism whereby CA maintains intestinal homeostasis.

## Materials and Methods

### Patients and Samples

This study was conducted in accordance with the Declaration of Helsinki and approved by the Institutional Review Board for Clinical Research of Sichuan Provincial People's Hospital (No.201685). Participants enrolled in the current study were well-informed and signed an informed consent before participation. Patients with UC (*n* = 23) were all recruited from the Department of Gastroenterology, Sichuan Provincial People's Hospital (Chengdu, China). Mucosa biopsy tissues were collected during endoscopic examinations, immediately kept in RPMI-1640 medium supplemented with 10% fetal bovine serum (FBS) (FBS-RPMI 1640) and then sent to our laboratory for tissue culture. To examine the *ex vivo* effect of CA on human mucosal inflammation, we performed tissue culture as reported previously (12). Briefly, mucosa tissues (2–3 pieces from each patient) were incubated overnight in FBS-RPMI 1640 with CA (10 μM) at 37°C and 5% CO_2_. As controls, mucosa tissues from the same patients were incubated overnight in FBS-RPMI 1640 in the absence of CA. After overnight incubation, tissues were collected for mRNA analysis.

### Induction of Colitis and Treatment

All procedures were approved by the Animal Care and Use Committee at Sichuan Provincial People's Hospital and performed in accordance with National Institutes of Health guidelines for animal care and use. Male C57BL/6 mice (purchased from the Shanghai Model Organisms, Shanghai, China) were used at their age of 10–12 weeks. All mice were raised in our facility in a specific pathogens-free environment. DSS-induced colitis was established as reported previously (13). Briefly, mice were fed *ad lib* with 2.5% (w/v) DSS solution (36,000–50,000 Da, MP Biomedicals, LLC; Solon, OH, USA) for 7 days, followed by a 3-day recover period with drinking water. To evaluate the severity of colitis, the weight, disease activity index (DAI), stool consistency, and the presence of blood in stools were daily monitored during the modeling period as reported previously (14). The DAI was calculated as follows: normal stools = 0, soft stools = 1, soft stools and slight bleeding = 2, loose stools and slight bleeding = 3, watery diarrhea or loose stools and gross bleeding = 4. Mice with *ad lib* drinking water served as naïve controls (NC). For CA treatment, DSS-exposed mice were intraperitoneally administrated with CA (50 mg/kg/day, dissolved in DMSO) or DMSO (the same volume as CA solution) from day 0 to day 10. On day 10, all mice were sacrificed by cervical dislocation, and colon samples were collected for further analysis.

### Intestinal Intraepithelial Lymphocyte (IEL) and Lamina Propria Leukocyte (LPL) Isolation

After sacrificing the mice, the whole gastrointestinal tract was immediately removed and mesenteric fat were carefully dissected. The colon was opened longitudinally and rinsed by phosphate buffered saline (PBS) to remove luminal content, which were then cut into 5–10 mm pieces. The pieces were incubated in 30 ml EDTA solution (5 mM EDTA, 10% FBS in Ca^2+^/Mg^2+^ free PBS) with continuous brisk stirring for 30 min at 37°C. The supernatants were filtered rapidly by a 70 μm cell strainer to remove cellular debris, which were centrifuged at 1,500 rpm at 4°C for 8 min to collect intestinal epithelial cells (IEC) and IEL. The residual tissues were collected for LPL isolation as reported previously (12). To purify IEL from IEC, cell pellets were resuspended in 4 ml of 40% percoll solution and layered onto 2 ml of 80% percoll solution, followed by gradient separation (centrifuge at 2,000 rpm at room temperature for 20 min). IEL were collected at the 40/80% interface, which were washed three times in FBS-RPMI 1640 for subsequent analysis. To obtain LPL, the residual tissues from the EDTA step were minced into smaller fragments (~0.5 mm) and incubated in 10 ml digestion solution, which contained type IV collagenase (1 mg/ml, Sigma Aldrich, Burlington, MA, USA) and FBS-RPMI 1640, in a shaking incubator at 37°C for 30 min. After incubation, the supernatants were passed through a 70 μM cell filter. Percoll gradient (40%/80%) separation of LPL was performed as mentioned above.

### Intestinal Permeability Assay

Intestinal barrier function was measured as previously described (15). Briefly, mice were deprived of food and water for 6–8 h, and then gavaged with FD-4 (FITC conjugated Dextran, FITC-Dextran, Sigma-Aldrich) at the dose of 0.5 mg/kg body weight. After 4 h, blood samples were collected by cardiac puncture and the fluorescence intensity in sera was measured (excitation, 485 nm; emission, 530 nm). The FD-4 concentration is determined by the standard curve produced by the dilution of FD-4 series. Quantitative colorimetric Limulus amebocyte Lysate (LAL) QCL-1000 assay kit (LONZA) was used to detect serum LPS concentrations according to the manufacturer's protocol.

### Flow Cytometry

Flow cytometry was performed as described previously (12, 13, 16). Briefly, 10^6^ cells were counted and collected in a FACS tube (5 ml) for each flow cytometric test. For surface staining, cells were incubated in dark with 20 μL Staining Buffer (BD Bioscience, San Diego, CA, USA) containing a viability dye (LIVE/DEAD™ Fixable Near-IR Dead Cell Stain Kit, Invitrogen, Thermo Fisher Scientific, USA) and indicated fluorochrome-conjugated antibodies for 30 min at 4°C. For intracellular cytokine staining, cells were pre-stimulated with a Cell Activation Cocktail (with Brefeldin A) (BioLegend, San Diego, CA, USA) for 5 h. After harvesting, cells were used for surface staining as mentioned above. Subsequently, the cells were fixed and permeabilized using a Cyto-Fast™ Fix/Perm Buffer Set (BioLegend), and fluorochrome-conjugated anti-mouse cytokine antibodies were added to stain the intracellular cytokines. A BD FACSCanto II was used to acquire the flow cytometric data, which were then analyzed with FlowJo version 10 for Windows (Tree Star, Ashland, OR, USA).

### Immunofluorescence

As reported previously (17), immunofluorescence assay was performed on 6 μm-thick frozen sections of colon tissue. Slides were baked for 30 min at 37°C. Next, the colonic sections were washed 3 times for 5 min by PBS. Cryosections (6 μm) were blocked in 5% BSA, 0.5% Triton × 100 in PBS for 2 h at room temperature. The sections were then incubated overnight at 4°C with indicated primary antibodies. When the incubation was completed, the slides were washed three times for 6 min using PBS. Secondary antibodies were added and incubated with colonic sections at room temperature for 2 h. The slides were sealed with neutral resin, and then photographed for analysis.

### Hematoxylin and Eosin (H&E) and Periodic Acid-Schiff (PAS) Staining

Dissected colon tissues were fixed with 4% paraformaldehyde (PFA) for 48 h, and then dehydrated using an automatic tissue processor and embedded in paraffin blocks. Paraffin blocks were cut into sections (4 μm) with a microtome (Leica), which were placed on slides for staining. After dewaxing, the sections were stained according to the instructions of hematoxylin and eosin (H-E) staining kit (Servicebio, Wuhan, China). The colonic goblet cells were labeled by alcian blue pas (AB-PAS) staining. After dewaxing and hydration, the slides were incubated in AB for 20 min, and then were washed with water, followed by incubation in 1% periodic acid for 10 min. After the appellate step, the slides were incubated in Schiff's reagent for 10 min. The slides were finally re-stained with hematoxylin for 30 s, washed and dehydrated, and then fixed with Pertex. The stained sections were evaluated and observed under optical microscope (Leica).

### Quantitative Real-Time PCR (qRT-PCR)

As discribed previously (12, 18), total RNA was extracted from indicated samples using TRIzol reagent (TransGen Biotech, China) according to the manufacturer's instructions. The Reverse Transcription Reaction System (TransGen Biotech) was used to Reverse transcribe was performed with 1μg of total RNA. For qRT-PCR analysis, the SYBR Green Kit (TaKaRa, Dalian, China) was applied to amplified cDNA according to the manufacturer's protocol. qRT-PCR detection was performed on a CFX96 Real-time RT-PCR system (Bio-Rad). Relative gene expression was normalized to endogenous GAPDH.

### Statistical Analysis

Statistical analyses were carried out with GraphPad Prism version 8.4 for Windows (GraphPad Software, San Diego, CA, USA). Unpaired Student's *t*-test (two-tailed) was applied for comparison between two groups were analyzed, and one-way analysis of variance (ANOVA) followed by Tukey's multiple comparisons test was applied to analyze differences among three or more groups. Statistical significance was set at ^*^P < 0.05.

## Results

### CA Alleviates DSS-Induced Colitis

**Figure 1:**
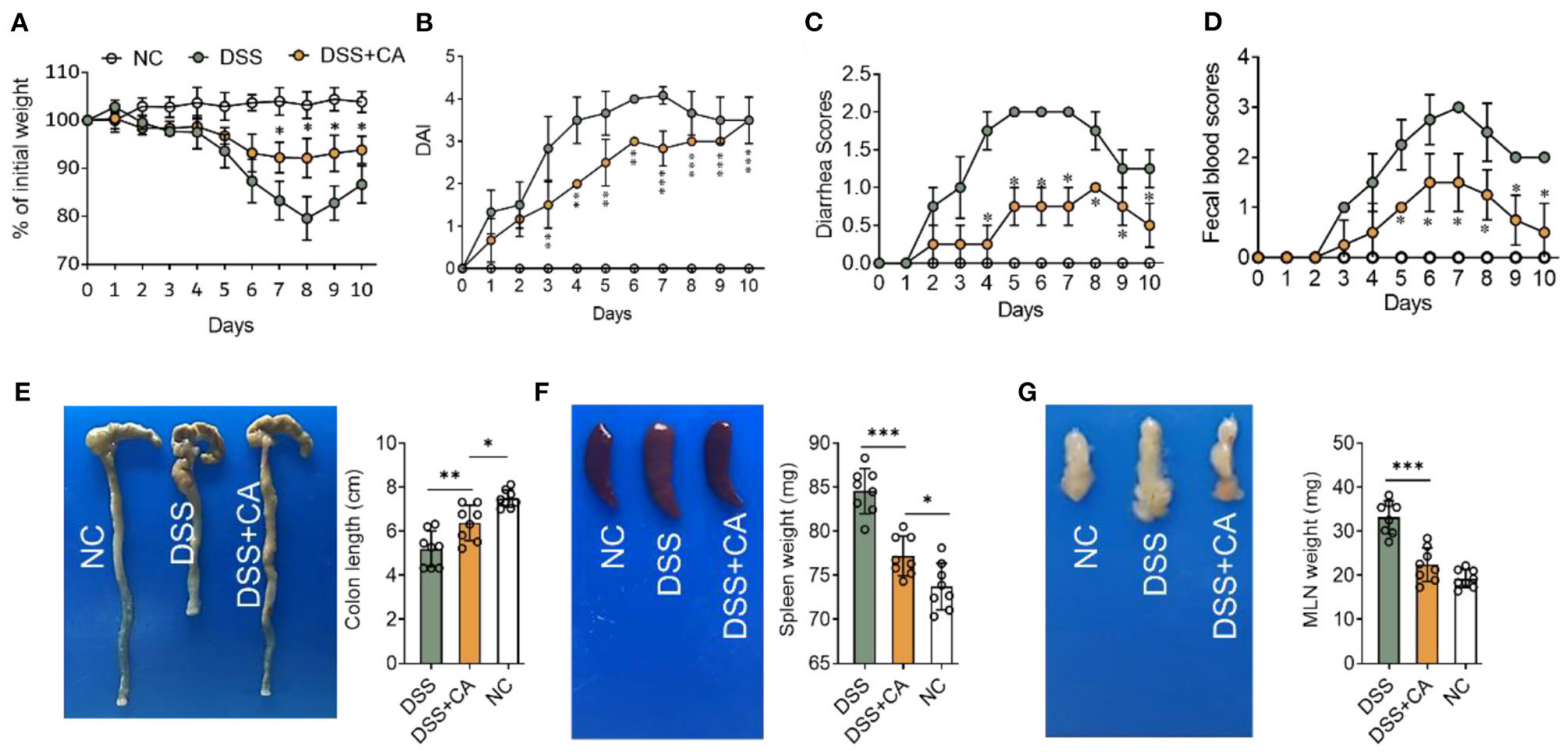
Carnosol (CA) alleviates the clinical signs in mice with DSS-induced colitis. Experimental colitis was induced by DSS (exposure to DSS for 7 days, followed by 3 days of recovery). Mice were fed with DSS (2.5%, w/v) in their drinking water with or without daily intraperitoneally with CA, while naïve control (NC) mice received regular drinking water. **(A)** Body weight changes following DSS induction of colitis. Data were recorded as a percentage of the initial body weight. **(B)** The DAI scores were recorded every day. The DAI scores were calculated according to weight loss, diarrhea and rectal bleeding. **(C)** Diarrhea was scored as follows: normal stool = 0, soft = 1, and watery = 2. **(D)** Rectal bleeding scores were determined as follows: no bleeding in stool = 0, blood traces in stool visible = 1, gross rectal bleeding = 3. Data are expressed as mean ± SD. **(A**–**D)** DSS group: *n* = 8, DSS+CA group: *n* = 8, NC group: *n* = 8. **(E)** Representative colon photos (left) and quantification of colon length (cm) of 3 groups (right). Colon length comparisons were shown in histogram. Representative gross morphology of spleens (**F**, left) and lymph nodes (**G**, left). Quantitative analysis of the weight (mg) of spleens (**F**, right) and lymph nodes (**G**, right). One-way analysis of variance (ANOVA) followed by Tukey's multiple comparisons test, *p < 0.05, **p < 0.01, and ***p < 0.001.

**Figure 2:**
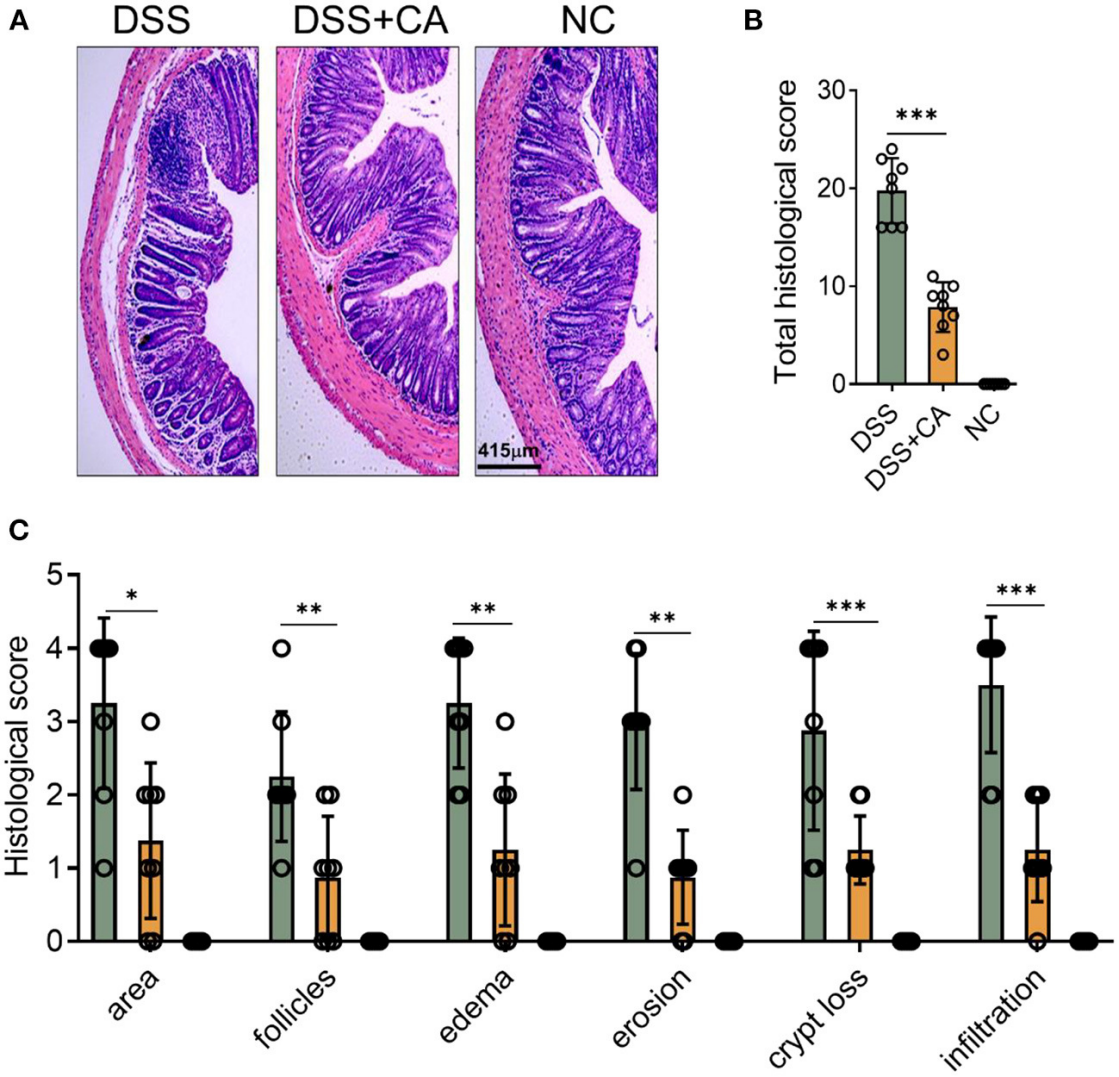
CA alleviates pathological alterations in DSS-induced colitis. Colitis was induced as described in Figure 1 and colon samples were collected on day 10. **(A)** Histological analysis of colonic sections. Representative images of H-E-stained colonic sections of each group. **(B)** Total histological scores were the sum of all sub-scores shown in **(C)**. **(C)** Colonic sections were scored on a scale of 0–4 based on the percentage of colon involvement by inflammation, percentage of crypt loss, presence of lymphoid follicles, edema, erosions, and density of infiltrating inflammatory cells. Data are presented as mean ± SD. Representative data of three independent experiments are shown. One-way analysis of variance (ANOVA) followed by Tukey's multiple comparisons test, *p < 0.05, **p < 0.01, and ***p < 0.001.

To examine whether administration of CA could alleviate intestinal mucosal inflammation, colitis was induced in mice by oral treatment of DSS, and CA was injected intraperitoneally from on day 0 to day 10. The clinical signs of colitis were monitored daily, including body weight, stool consistency and rectal bleeding. CA significantly alleviated the weight loss (Figure 1A) induced by DSS administration and reduced the disease activity index (Figure 1B), diarrhea (Figure 1C) and fecal blood scores (Figure 1D) during the progression of colitis, suggesting a protective role of CA in experimental colitis. On day 10, mice were sacrificed and CA attenuated the colonic shortening (Figure 1E), splenomegaly (Figure 1F), and mesenteric lymphadenopathy (Figure 1G) induced by DSS, compared with DSS-exposed mice without CA. To further explore the role of CA in mucosal inflammation and damage, H-E staining and evaluation were performed on colonic sections in different groups. Immune infiltrate, flattening of the glands, disruption to the epithelial lining, destruction of mucosal epithelium, and goblet cell loss (Figure 2A) were observed in colon tissues from DSS mice. CA treatment significantly relived mucosal damage in DSS mice. No significant mucosal damage was observed in NC mice. As shown in Figures 2B,C, DSS mice exhibited a markedly higher pathology score (19), characterized by increased epithelial disruption, follicle aggregation, enhanced erosion, increased crypt loss and increased infiltration of immune cells compared to CA-treated DSS mice. In addition, CA treatment could ameliorate symptoms of on-going DSS-induced colitis (Supplementary Figure 1).

### CA Modulates Mucosal Immune Cell Infiltration and Inflammatory Responses

**Figure 3:**
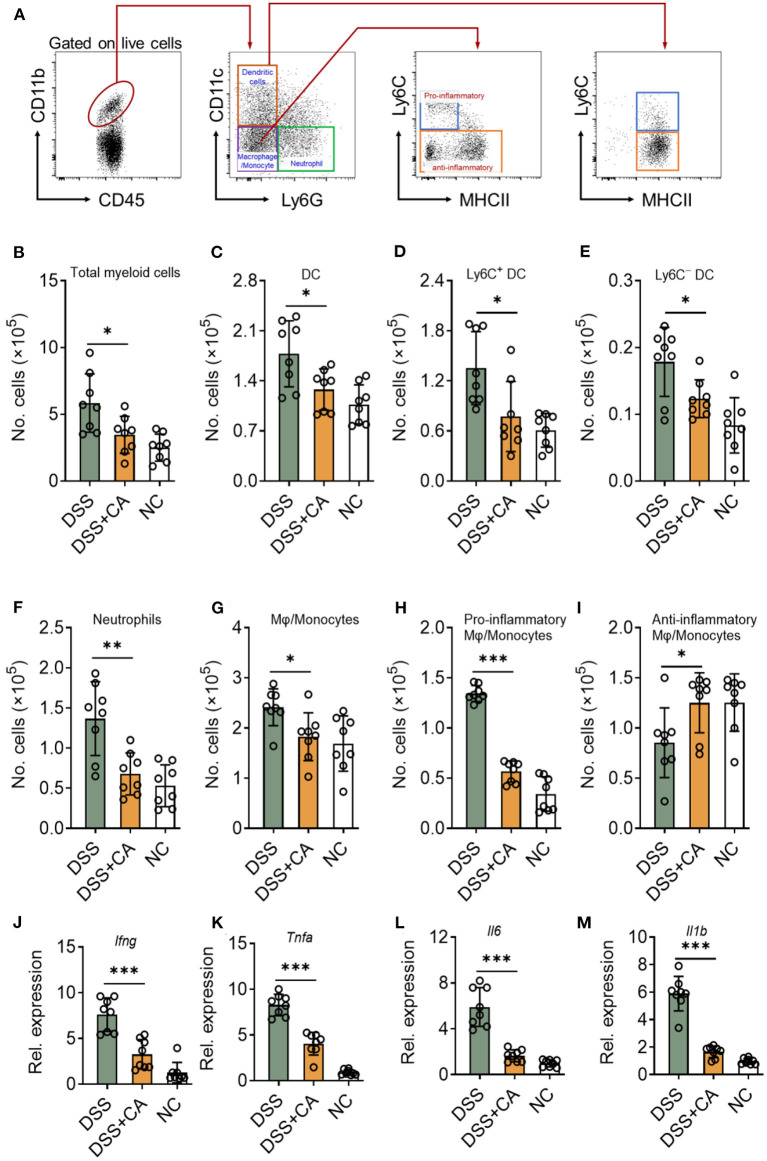
CA suppresses colonic inflammatory responses in DSS-treated mice. Colitis was induced as described in Figure 1 and colon lamina propria leukocyte (LPL) were isolated on day 10. **(A)** Gating strategy for lamina propria myeloid cells. Different cell populations were identified as follows: total myeloid cells (CD45^+^ CD11b^+^), dendritic cells (DC, CD45^+^ CD11b^+^ Ly6G^−^ CD11c^+^), neutrophils (CD45^+^ CD11b^+^ Ly6G^+^), monocytes/macrophages (CD45^+^ CD11b^+^ CD11c^−^ Ly6G^−^), Ly6C^+^ DC (CD45^+^ CD11b^+^ Ly6G^−^ CD11c^+^ Ly6C^+^), Ly6C^−^ DC (CD45^+^ CD11b^+^ Ly6G^−^ CD11c^+^ Ly6C^−^), total myeloid cells (CD45^+^ CD11b^+^), pro-inflammatory monocytes/macrophages (CD45^+^ CD11b^+^ CD11c^−^ Ly6G^−^ Ly6C^+^ MHC II^−^), and anti-inflammatory monocytes/macrophages (CD45^+^ CD11b^+^ CD11c^−^ Ly6G^−^ Ly6C^−^). Quantification of different cell populations are shown in **(B–I)**. **(J–M)** The expression levels of indicated inflammatory agents in colons were determined by qRT-PCR. Dates are presented as mean ± SD. Representative data of three independent experiments are shown. One-way analysis of variance (ANOVA) followed by Tukey's multiple comparisons test, *p < 0.05, **p < 0.01, and ***p < 0.001.

Since DSS-induced colitis was characterized by the of immune cell infiltration (especially innate immune cells) and proinflammatory cytokine accumulation (20), we next sought to investigate whether CA could suppress DSS-induced mucosal inflammatory responses in mice. To this end, we first looked at the number of innate cells in the lamina propria using our previously established flow cytometric gating strategy (Figure 3A) (21). Compared with CA-treated DSS mice, flow cytometric staining for CD45 and CD11b showed a significant increase in CD45^+^ CD11b^+^ cells (total myeloid cells) in the lamina propria of DSS mice (Figure 3B). We employed CD11c and Ly6G to identify dendritic cells (DC) and neutrophils from total myeloid cells. CA significantly decreased the number of total DC (Figure 3C) and their subsets (Figures 3D,E), which has been reported to be associated with intestinal inflammation (22). Neutrophil infiltration is a hallmark of human UC and mouse DSS-induced colitis (23). We found Ly6G^+^ neutrophil accumulation was evidently lower in CA-treated DSS mice than DSS counterparts (Figure 3F). In addition, we checked the monocyte/macrophage population (CD11b^+^ CD11c^−^ Ly6G^−^), and found that CA significantly alleviated monocyte/macrophage infiltration in the lamina propria of DSS mice (Figure 3G). Moreover, we analyzed the phenotype of monocytes/macrophages by using additional markers (Ly6C and MHC II) (24, 25) and found that the number of pro-inflammatory monocytes/macrophages (Ly6C^+^ MHCII^−^) was significantly decreased (Figure 3H) but the anti-inflammatory subset (Ly6C^−^) was up-regulated (Figure 3I) in CA DSS mice than DSS mice. Given overexpression of pro-inflammatory cytokines was a hallmark of experimental colitis, we then analyzed the effect of CA on colonic mucosal cytokine expression in colitis. As illustrated in Figures 3J–M, colonic TNF-α, IL-1β, IL-6, and IFN-γ expression was significantly increased by DSS, which was suppressed by CA treatment. Collectively, CA could inhibit mucosal immune cell infiltration and inflammatory responses during DSS-induced colitis.

### CA Restores Epithelial Barrier Functions During Colitis

**Figure 4:**
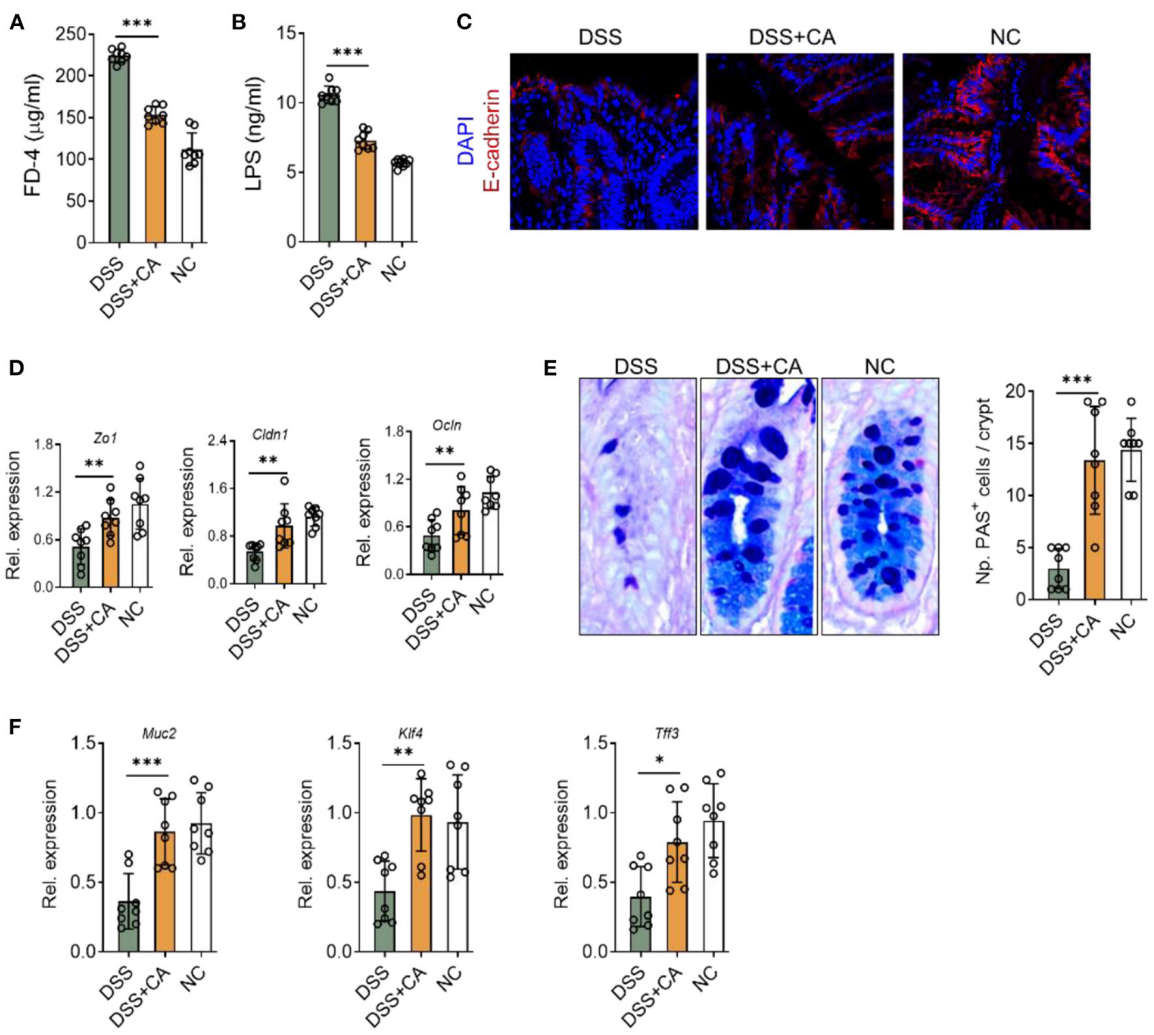
CA restores epithelial barrier functions in intestinal inflammation. Colitis was induced as described in Figure 1. **(A**,**B)** Intestinal permeability in all three groups was assessed on day 10. **(A)** Serum FD-4 levels were measured 4 h after oral gavage of FD-4. **(B)** Levels of serum lipopolysaccharides (LPS) were measured. **(C)** Colonic sections were stained for E-cadherin and DAPI. Representative immunofluorescence images are shown. **(D)** Expression levels of colonic ZO-1, claudin-1, and occludin in all three groups by qRT-PCR. **(E)** Colonic goblet cells were identified by PAS staining. Representative images are shown (left) and the number of goblet cells was quantified. **(F)** The expression of colonic MUC2, KLF4, and TFF3 were detected by qRT-PCR. Data are presented as mean ± SD. Representative data of three independent experiments are shown. One-way analysis of variance (ANOVA) followed by Tukey's multiple comparisons test, *p < 0.05, **p < 0.01, and ***p < 0.001.

We next explored whether CA affect intestinal barrier functions during colitis. Firstly, we found that DSS insults led to a breakdown of epithelial barrier, identified by an increase in serum levels of FD-4 (Figure 4A) and LPS (Figure 4B). We performed immunofluorescent staining for E-cadherin, which is an essential component of epithelial tight junctions. We found severe loss of E-cadherin in DSS mice, and CA potently restored E-cadherin expression during colitis (Figure 4C), suggesting that CA could protect epithelial tight junctions from breakdown induced by DSS. This observation was also evidenced by mRNA analysis of ZO-1, claudin-1, and occludin expression (Figure 4D). In addition, since goblet cells contribute majorly to the epithelial barrier homeostasis and goblet impairment is a critical hallmark of UC (26), we explored the role of CA on goblet cells during colitis. As shown in Figure 4E, CA remarkably reversed DSS-induced decreases of mucus-producing goblet cells in the colon, which further confirmed by qRT-PCR analysis (Figure 4F). Taken together, CA is found to be protective for epithelial barrier function during colitis.

### CA Restores IEL Homeostasis During DSS-Induced Colitis

**Figure 5:**
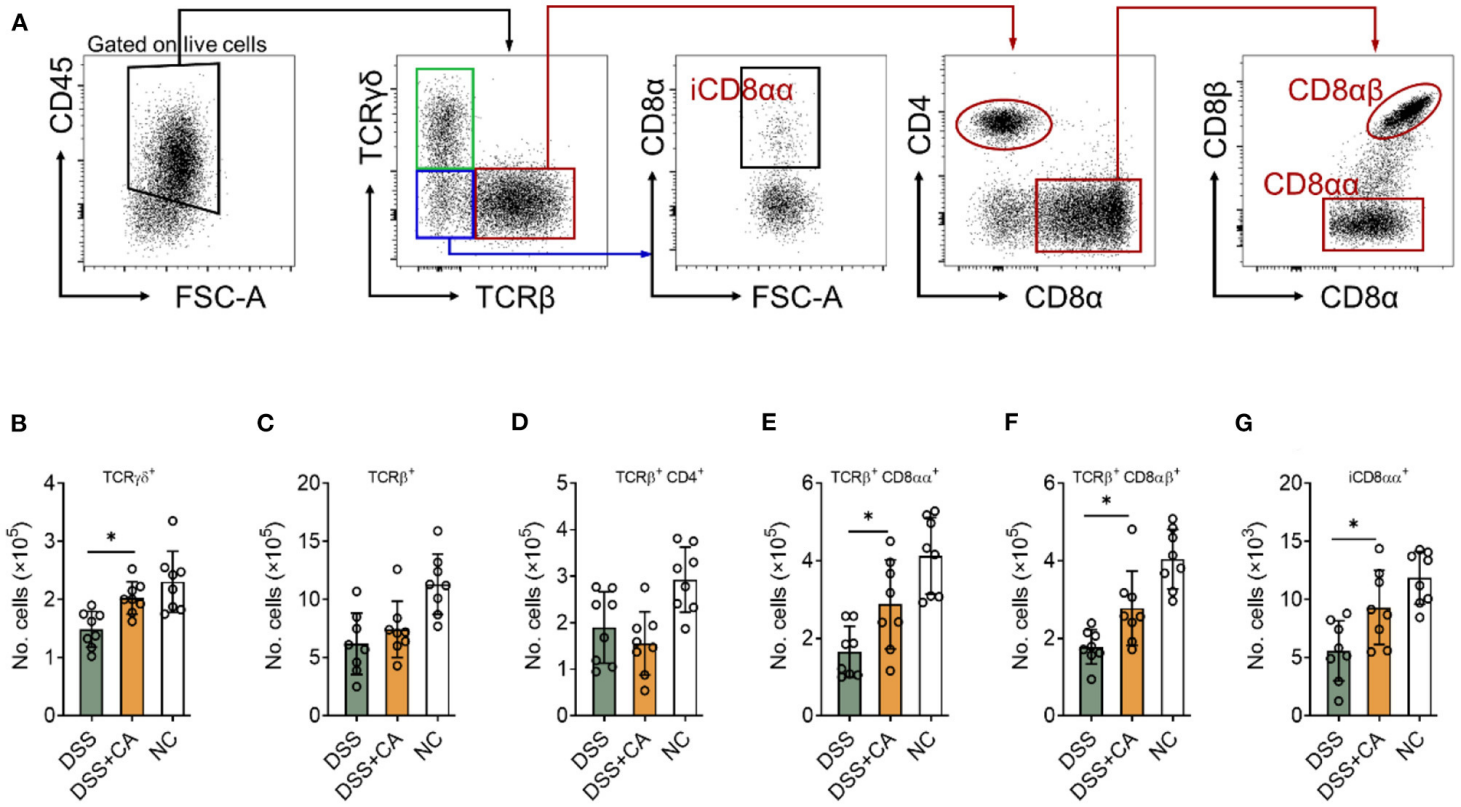
CA restores intestinal intraepithelial lymphocyte (IEL) homeostasis during DSS-induced colitis. Colitis was induced as described in Figure 1 and IEL were isolated on day 10. Flow cytometry was performed to determine IEL subpopulations. **(A)** Gating strategy. **(B–G)** Quantification of **(B)** TCRγδ^+^, **(C)** TCRβ^+^, **(D)** TCRβ^+^ CD4^+^, **(E)** TCRβ^+^ CD8αα^+^, **(F)** TCRβ^+^ CD8αβ^+^, and **(G)** iCD8αα^+^ subpopulations are shown. Data are presented as mean ± SD. Representative data of three independent experiments are shown. One-way analysis of variance (ANOVA) followed by Tukey's multiple comparisons test, *p < 0.05.

Intestinal epithelial lymphocytes (IEL) are essential components of the epithelial barrier, which can rapidly respond to pathogen antigens and play a major role to maintain intestinal barrier functions. Dysregulation of IEL subpopulations contributes to mucosal inflammation. To determine the role of CA on IEL during DSS colitis, we isolated IEL from the colon and performed flow cytometry to evaluate IEL subpopulations (Figure 5A shows the gating strategy) (27). CA-treated DSS mice showed significantly higher number of TCRγδ^+^ (Figure 5B), TCRγδ^+^ CD8αα^+^ (Figure 5E), TCRβ^+^ CD8αβ^+^ (Figure 5F), and TCRγδ^−^ TCRβ^−^ CD8αα^+^ (Figure 5G) IEL in comparison with their DSS counterparts. Although CA-treated DSS mice showed higher number of total TCRβ^+^ IEL and lower number of TCRβ^+^ CD4^+^ IEL than DSS mice, the differences didn't reach a statistical significance (Figures 5C,D). These data suggest that CA can restore IEL homeostasis in mice with DSS-induced colitis.

### CA Suppresses ER Stress in IEC During DSS-Induced Colitis

**Figure 6:**
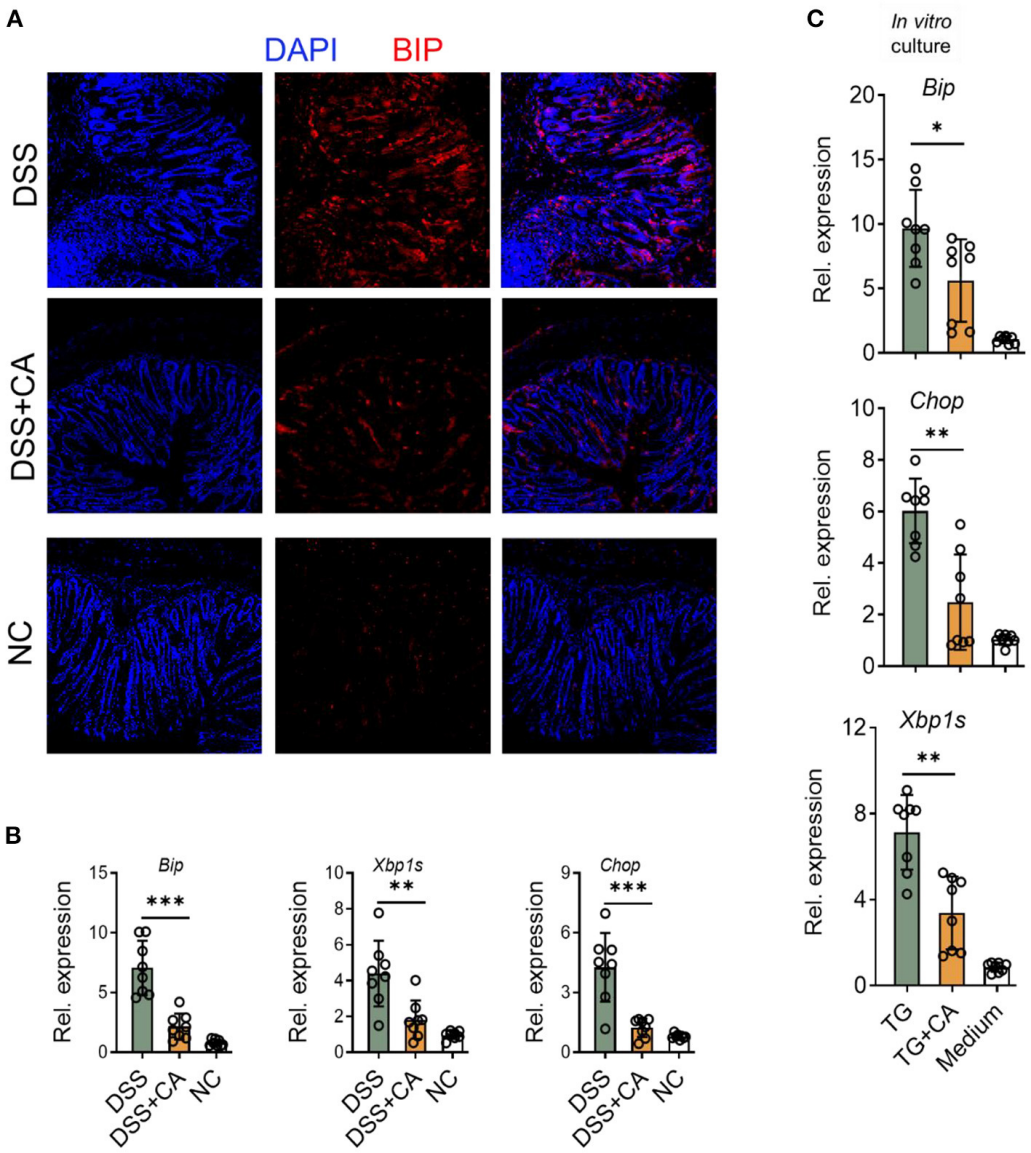
CA suppresses ER stress in IEC. Colitis was induced as described in Figure 1 and colon samples were collected on day 10. **(A)** Colonic sections were stained for BIP and DAPI. Representative immunofluorescence images are shown. **(B)** The expression levels of BIP, XBP1s, and CHOP were determined by qRT-PCR. **(C)** HCT-116 cells (an intestinal epithelial cell line) were incubated overnight with Thapsigargin (TG, 100 nM) in the presence or absence of CA (10 μM). The expression levels of BIP, CHOP, and XBP1s were determined by qRT-PCR. Data are presented as mean ± SD. Representative data of three independent experiments are shown. One-way analysis of variance (ANOVA) followed by Tukey's multiple comparisons test, *p < 0.05, **p < 0.01, and ***p < 0.001.

Given growing evidence have suggested that inflammation-induced ER stress responses could compromise epithelial barrier functions and deteriorate intestinal inflammation IBD (28). Therefore, we asked whether the anti-colitic and barrier-protective effects of CA were associated with a decrease of molecular features of ER stress responses in the colonic mucosa. Expectedly, we found that CA greatly reduced epithelial expression of Bip (Figure 6A), a feature marker of ER stress, which was strongly induced in colitis. qRT-PCR was performed to analyze molecular features of ER stress responses and showed that CA inhibited IEC expression of Bip, CHOP, and XBP1s, which were all induced by DSS (Figure 6B). Additionally, we aimed to determine whether CA indeed regulated ER stress signaling. To this end, HCT-116 cells were cultured *in vitro* with an ER-stress inducer (thapsigargin), which induced ER stress, indicated by increased expression of Bip, CHOP, and XBP1s. Thapsigargin-induced ER stress was significantly suppressed by CA (Figure 6C). Taken together, our data suggest that CA maintains intestinal barrier functions, probably via inhibiting ER stress.

### CA Reduces Inflammatory Responses and ER Stress in Mucosal Tissues of UC Patients

**Figure 7:**
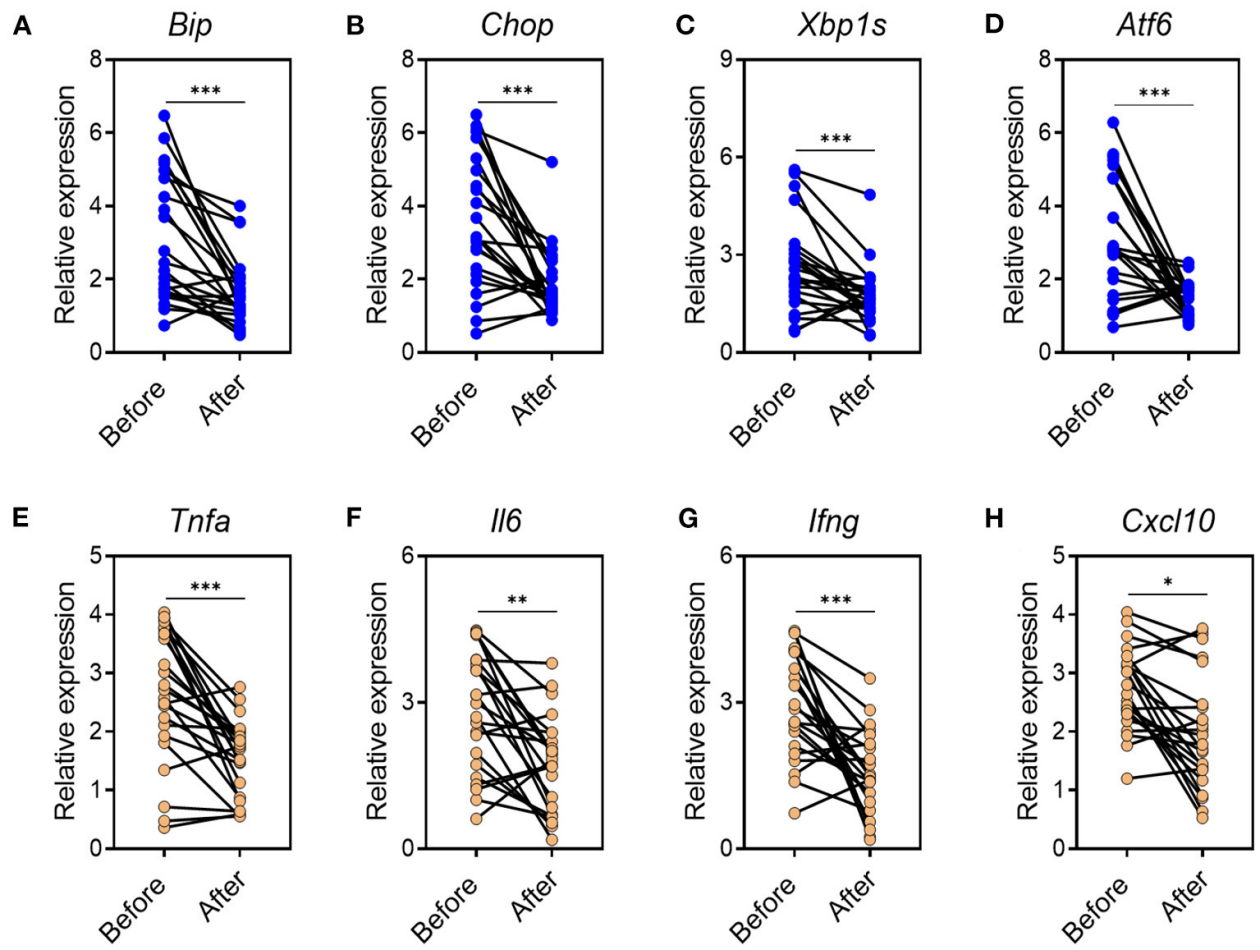
CA reduces inflammatory responses and ER stress in mucosal tissues of UC patients. Inflamed mucosal tissues were obtained from patients with UC (*n* = 23) as indicated and incubated overnight with or without CA (10 μ M). qRT-PCR was performed for gene expression. **(A–D)** ER stress signaling gene expression (**A**, BIP; **B**, CHOP; **C**, XBP1s; **D**, ATF6) and **(E–H)** inflammatory agent expression (**E**, TNF-a; **F**, IL-6; **G**, IFN-g; **H**, CXCL10) are shown. Data are presented as mean ± SD. Unpaired Student's *t* test (two-tailed), *p < 0.05, **p < 0.01, and ***p < 0.001.

Based on the data found in mouse model, we next investigated whether CA regulated mucosal inflammatory responses and ER stress in human UC. To this end, we collected inflamed mucosal biopsies from 23 patients with UC, and cultured overnight with CA. Tissues cultured in medium alone served as controls. After overnight incubation, tissues were harvested and qRT-PCR was performed to analyze cytokine and ER stress signaling molecule expression. Incubation with CA resulted in significantly reduced expression of ER stress signaling molecule (Figures 7A–D) and pro-inflammatory agents (Figures 7E–H). Collectively, our data demonstrated that CA exerts a crucial anti-inflammatory function in human UC and mouse colitis.

## Discussion

Although the role of adaptive immunity in UC has been noted, epithelial barrier is the first defense line against luminal pathogens through forming a physical barrier, so that they exert essential function in maintaining intestinal homeostasis (29). It is reported that impaired IEC barrier function may be one of the important causes of IBD (30). In recent years, many studies have focused on the value of natural metabolites to prevent and cure diseases. Advances in biological techniques has enhanced the understanding of the characteristics and properties of natural plant products, as well as their therapeutic potential. However, the composition of natural metabolites is complex and pharmacologically active moieties need further exploration. Therefore, an in-depth understanding of the nature of biologically active compounds as well as their pharmacological function can further enhance their utility in diseases, such as IBD. In the current study, we established a mouse experimental colitis, which mimicks human UC. In this model, we investigated the effect of CA on intestinal inflammation and epithelial barrier functions. We found that: (1) CA significantly alleviates mucosal injuries caused by DSS; (2) lumina propria immune cell infiltration was efficiently reduced by CA in DSS mice; (3) CA maintained epithelial barrier functions during colitis; (4) CA suppresses ER stress in IEC; (5) The immunoregulatory effects of CA were also observed on human UC mucosa samples. Our findings provide new evidences that CA might be a promising candidate as a therapeutic cure for IBD.

Intestinal epithelial cells (IEC) are the first line protecting the host from luminal antigens and includes several cell types, such as enterocytes, Paneth cells, and goblet cells (31). Enterocytes, also called intestinal absorptive cells, constitutes a barrier through cell-cell adhesion, which allows a very limited passage of material in both directions. Furthermore, mucus produced by goblet cells together with antimicrobial agents secreted by Paneth cells forms another physical barrier against intestinal antigens. Growing evidences have demonstrated that IEC play a crucial role in maintaining intestinal homeostasis, and compromised barrier function has been indicated in the initiation of intestinal mucosal inflammation. Beyond that, disruption of intestinal epithelial barrier is also able to cause systemic immune activation. The interactions between microbiota and the host occur at the surface of IEC, which contribute to a variety of diseases at a broad range of extra-intestinal sites, including primary sclerosing cholangitis, multiple sclerosis, and type 1 diabetes (29, 32). Therefore, a full-functional IEC barrier is essential for maintaining mucosal immune homeostasis of the host and comprehensively understanding the regulation of their properties is benefit of developing new strategies to prevent and treat IBD. Here, we demonstrate CA protects epithelial barrier functions by several means, including maintaining tight junction integrity, reversing inflammatory-induced decrease of goblet cells, and restoring IEL homeostasis, suggesting a critical role of CA in mucosal homeostasis.

IEL represent one of the largest immunological populations in the body, which reside within the IEC monolayer and cooperate with IEC to maintain the barrier functions (33). The basis of TCR expression, IEL can be classified into two major subpopulations: IEL expressing TCR and TCR-negative IEL. The former subpopulation can be further divided into TCRαβ^+^ CD4^+^, TCRαβ^+^ CD8αα^+^, TCRαβ^+^ CD8αβ^+^, and TCRγδ^+^ cells. The TCR-negative subpopulation includes innate lymphoid cells (ILC) and T cells containing intracellular CD3γ-chains (iCD3^+^). A subset of iCD3^+^ T cells expresses CD8αα, which is named as iCD8α^+^ (34). Due to their anatomical location, IEL play a critical role as sentinels surveilling luminal microbial and food antigens, providing protection against potential pathogen invasion (35). To exert their immunological functions, IEL need to maintain their homeostasis. However, since IEL population are heterogenic, requirements for their homeostasis largely depend on the particular type of IEL. It has been demonstrated that TCRγδ^+^ cells are responsible for pathogen surveillance, tissue repair, and barrier protection (36). iCD8α cells can protect against necrotizing enterocolitis by fighting against *Citrobacter rodentium* (37). TCRαβ^+^ CD8αα^+^ IEL can ameliorate experimental colitis, showing an immunoregulatory and protective role in mucosal homeostasis (38). Our results in this study reveal that CA is critical for the homeostasis of several IEL subpopulations (TCRγδ^+^, TCRαβ^+^ CD8αα^+^, TCRαβ^+^ CD8αβ^+^, and iCD8αα^+^). These findings suggest that CA treatment ensures proper homeostasis of most types of IEL, thus maintaining intestinal epithelial barrier functions.

The importance of IEC in intestinal homeostasis has been evidenced by numerous animal studies and genome-wide association studies, in which a number of genes were identified to be tightly associated with IEC functions. Cellular stress signaling such as ER stress has been linked to the proliferation, differentiation, apoptosis, and normal functions of different subsets of IEC in the gut (39). Multiple molecular mechanisms have been reported regarding how IEC ER stress induces mucosal inflammation (28), including (1) ER stress causes epithelial cell death, which directly destructs the barrier; (2) ER stress impairs goblet cell and Paneth cell functions, resulting in compromised antimicrobial peptide and mucin secretion; (3) ER stress triggers inflammatory signaling in IEC. Additionally, patients with UC exhibit up-regulated ER stress in IEC located in inflammatory mucosa (40). Considering the importance of ER homeostasis in IEC, it has been suggested that epithelial ER stress might be a promising therapeutic target for UC. For example, glutamine, as an energy source for colonocytes, was found to be a potent ER stress suppressor. Glutamine treatment significantly ameliorated experimental colitis in rats (41). Salubrinal, a recently identified inhibitor of eIF2α dephosphorylation, is capable to protect against ER stress in various model systems. Salubrinal has been shown to relief colonic inflammation in mice with deficiency in IL-10 and NADPH oxidase 1 or DSS-induced colitis (42, 43). Here, we demonstrate that CA is able to suppress colitis-induced ER stress in IEC, revealing potential mechanisms whereby CA maintains epithelial barrier functions and protects against DSS-induced colitis in mice.

In summary, we here revealed that CA, a natural active ortho-diphenol diterpene compound, acted as an effective drug for experimental colitis, which maintains proper epithelial barrier functions, probably via suppressing IEC ER stress. Our findings provide new evidence that CA might be a promising candidate as a therapeutic cure for IBD.

## Data Availability Statement

The original contributions presented in the study are included in the article/Supplementary Material, further inquiries can be directed to the corresponding author/s.

## Ethics Statement

The studies involving human participants were reviewed and approved by the Institutional Review Board for Clinical Research of Sichuan Provincial People's Hospital. The patients/participants provided their written informed consent to participate in this study. The animal study was reviewed and approved by the Animal Care and Use Committee at Sichuan Provincial People's Hospital.

## Author Contributions

FL and CH conceptualized and designed the study plan and edited the manuscript. XX, GZ, KP, and YG conducted the experiments. CG diagnosed the patients and collected the clinical samples. FL, CH, XX, and GZ analyzed the data and prepared the original draft. All authors discussed and revised the manuscript and agreed to the published version of the manuscript.

## Funding

This work was financially supported by grants from the National Natural Science Foundation of China (82070985) and Foundation of Sichuan Science and Technology Department (2021JDJQ0044).

## Conflict of Interest

The authors declare that the research was conducted in the absence of any commercial or financial relationships that could be construed as a potential conflict of interest.

## Publisher's Note

All claims expressed in this article are solely those of the authors and do not necessarily represent those of their affiliated organizations, or those of the publisher, the editors and the reviewers. Any product that may be evaluated in this article, or claim that may be made by its manufacturer, is not guaranteed or endorsed by the publisher.
