# Supplementary material for: Carnosol Maintains Intestinal Barrier Function and Mucosal Immune Homeostasis in DSS-Induced Colitis
Source: Front Nutr. 2022 May 24;9:894307. doi: 10.3389/fnut.2022.894307 (PMC9172907; doi:10.3389/fnut.2022.894307)
Supplement: Supplementary file 1 [file Image_1.pdf]

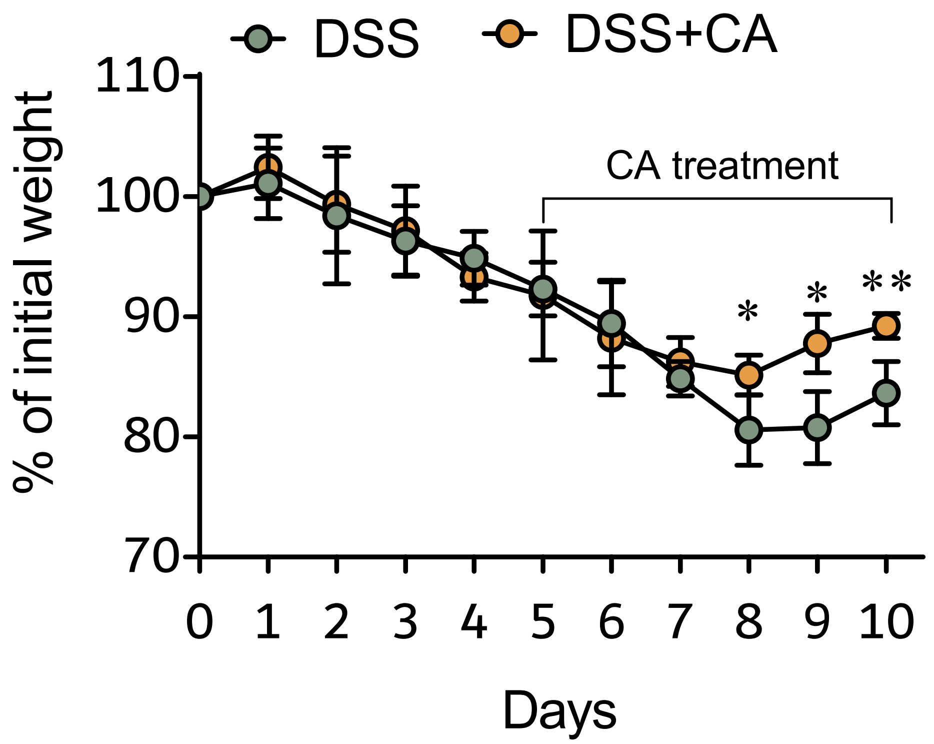

Supplementary Figure 1 Carnosol (CA) ameliorates weight loss of mice with on-going DSS-induced colitis. Experimental colitis was induced by DSS (exposure to DSS for 7 days, followed by 3 days of recovery), mice were fed with DSS (2.5%, w/v) in their drinking water. One group of DSS mice were treated with CA treatment from day 5 to 10. Body weight changes following DSS induction of colitis. Data was recorded as a percentage of the initial body weight. Data are presented as mean ± SD. Unpaired Student's t test (two-tailed), $*p < 0.05$, $**p < 0.01$.
