# Supplementary material for: Carnosol Maintains Intestinal Barrier Function and Mucosal Immune Homeostasis in DSS-Induced Colitis
Source: Front Nutr. 2022 May 24;9:894307. doi: 10.3389/fnut.2022.894307 (PMC9172907; doi:10.3389/fnut.2022.894307)
Supplement: Supplementary file 2 [file Data_Sheet_1.docx]

**Supplementary Table. 1 Antibodies used for flow cytometry**

| **Antibody** | **Clone** | **Cat. No** |
| --- | --- | --- |
| FITC anti-mouse TCR γ/δ Antibody | UC7-13D5 | 107504 |
| FITC anti-mouse CD3 Antibody | 17A2 | 100204 |
| Alexa Fluor® 488 anti-mouse/human CD11b Antibody | M1/70 | 101207 |
| PerCP anti-mouse CD11c Antibody | N418 | 117326 |
| PerCP/Cyanine5.5 anti-mouse TCR β chain Antibody | H57-597 | 109228 |
| APC anti-mouse I-A/I-E Antibody | M5/114.15.2 | 107614 |
| APC anti-mouse CD8b.2 Antibody | 53-5.8 | 140410 |
| Alexa Fluor® 647 anti-mouse/rat/human FOXP3 Antibody | 150D | 320014 |
| Brilliant Violet 510™ anti-mouse CD8a Antibody | 53-6.7 | 100752 |
| Brilliant Violet 510™ anti-mouse F4/80 Antibody | BM8 | 123135 |
| Brilliant Violet 421™ anti-mouse CD4 Antibody | GK1.5 | 100443 |
| Brilliant Violet 421™ anti-mouse IL-17A Antibody | TC11-18H10.1 | 506926 |
| Pacific Blue™ anti-mouse CD45 Antibody | S18009F | 157212 |
| PE anti-mouse Ly-6C Antibody | HK1.4 | 128008 |
| PE anti-mouse CD3 Antibody | 17A2 | 100206 |
| PE anti-mouse IFN-γ Antibody | XMG1.2 | 505808 |
| PE/Cyanine7 anti-mouse Ly-6G Antibody | 1A8 | 127618 |
| PE/Cyanine7 anti-mouse CD4 Antibody | GK1.5 | 100422 |

**Supplementary Table. 2 Primers for qRT-PCR**

|  | Gene | Sequence (5' to 3') |
| --- | --- | --- |
| Mouse | BIP-Forward | ACTTGGGGACCACCTATTCCT |
|  | BIP-Reverse | ATCGCCAATCAGACGCTCC |
|  | CHOP-Forward | CTGGAAGCCTGGTATGAGGAT |
|  | CHOP--Reverse | CAGGGTCAAGAGTAGTGAAGGT |
|  | XBP1S-Forward | AGCAGCAAGTGGTGGATTTG |
|  | XBP1S-Reverse | GAGTTTTCTCCCGTAAAAGCTGA |
|  | ATF6-Forward | TGGAGCAGGATGTCCCGTT |
|  | ATF6-Reverse | CTGTGGAAAGATGTGAGGACTC |
|  | IL-1β-Forward | GCAACTGTTCCTGAACTCAACT |
|  | IL-1β -Reverse | ATCTTTTGGGGTCCGTCAACT |
|  | IL-6-Forward | TAGTCCTTCCTACCCCAATTTCC |
|  | IL-6-Reverse | TTGGTCCTTAGCCACTCCTTC |
|  | TNF-α-Forward | CCCTCACACTCAGATCATCTTCT |
|  | TNF-α-Reverse | GCTACGACGTGGGCTACAG |
|  | IFN-γ-Forward | ATGAACGCTACACACTGCATC |
|  | IFN-γ-Reverse | CCATCCTTTTGCCAGTTCCTC |
| Human | TNF-α-Forward | CCTCTCTCTAATCAGCCCTCTG |
|  | TNF-α-Reverse | GAGGACCTGGGAGTAGATGAG |
|  | IL-6-Forward | ACTCACCTCTTCAGAACGAATTG |
|  | IL-6-Reverse | CCATCTTTGGAAGGTTCAGGTTG |
|  | IFN-γ-Forward | TCGGTAACTGACTTGAATGTCCA |
|  | IFN-γ-Reverse | TCGCTTCCCTGTTTTAGCTGC |
|  | BIP-Forward | CACGGTCTTTGACGCCAAG |
|  | BIP-Reverse | CCAAATAAGCCTCAGCGGTTT |
|  | CHOP-Forward | GAACGGCTCAAGCAGGAAATC |
|  | CHOP--Reverse | TTCACCATTCGGTCAATCAGAG |
|  | ATF6-Forward | GGGAACTCCTGCCGATCTTC |
|  | ATF6-Reverse | TGTGGAGAGACGCGATGACT |
|  | CXCL10-Forward | GTGGCATTCAAGGAGTACCTC |
|  | CXCL10-Reverse | TGATGGCCTTCGATTCTGGATT |
|  | XBP1S-Forward | CCCTCCAGAACATCTCCCCAT |
|  | XBP1S-Reverse | ACATGACTGGGTCCAAGTTGT |
